# Editorial Note: Somatization symptomology and its association with stress in patients with irritable bowel syndrome

**DOI:** 10.1371/journal.pone.0331058

**Published:** 2025-08-26

**Authors:** The PLOS One Editors

This article [1] was identified as one of a series of submissions for which we have concerns that the peer review did not meet the expected standards for *PLOS One*. We regret that the issues were not identified and addressed prior to the article’s publication.

This note is not intended to question the quality of the article or contributions of the authors to the review process.

## References

[1] Akhlaq S, Kazmi N, Kazmi SMH, Atiq M, Hussain S, Bajwa A, et al. Somatization symptomology and its association with stress in patients with irritable bowel syndrome. PLoS One. 2025;20(1):e0312506. doi: 10.1371/journal.pone.0312506 39761297 PMC11703010

